# A Comparison of Surgeon Estimated Times and Actual Operative Times in Pediatric Dental Rehabilitation under General Anesthesia. A Retrospective Study

**DOI:** 10.3390/jcm12134493

**Published:** 2023-07-05

**Authors:** Faris A. Alotaibi, Mohammed M. Aljuaid

**Affiliations:** 1 Department of Pediatric Dentistry, King Saud Medical City, Riyadh 12746, Saudi Arabia; 2 Department of Health Administration, College of Business Administration, King Saud University, Riyadh 11587, Saudi Arabia; maljuaid@ksu.edu.sa

**Keywords:** dental rehabilitation, general anesthesia, estimated times, actual times

## Abstract

This retrospective study aimed to compare the accuracy of the pediatric dental surgeon’s estimated operative times for dental rehabilitation under general anesthesia (DRGA) in pediatric patients. This study population included 674 pediatric patients who underwent DRGA at the study facility between January 2022 and December 2022, using convenience sampling to select patients who met our inclusion criteria. Data were collected from electronic medical and anesthesia records based on several factors, including patient-related factors such as age and gender, surgeon-related factors such as rank and experience, and anesthesia-related factors such as induction and recovery time (in minutes). This study highlights a significant difference between the surgeon’s estimated time (SET) and actual operative time (AOT) for pediatric DRGA procedures, with a mean difference of 19.28 min (SD = 43.17, *p* < 0.0001), indicating a tendency for surgeons to overestimate surgery time. Surgical procedure time was the strongest predictor of this discrepancy, with an R square value of 0.427 and a significant *p*-value of 0.000. Experience with surgeons, anesthesia induction, and recovery time were also significant predictors. Meanwhile, age, gender, and rank of surgeons did not significantly predict the difference between SET and AOT. Therefore, the study suggests that surgeons should adjust their estimates for pediatric DRGA procedures, specifically emphasizing a more accurate estimation of surgery time, to ensure adequate resource allocation and patient outcomes.

## 1. Introduction

Dental rehabilitation under general anesthesia (DRGA) refers to a dental treatment procedure carried out under general anesthesia to enable the dentist to perform comprehensive dental work on a patient, particularly children whose cooperation is difficult to obtain during dental procedures. Due to the widespread prevalence of decayed deciduous teeth, DRGA has become increasingly popular, particularly for children’s dental treatment [1,2]. According to the World Health Organization (WHO), 532 million children worldwide have permanent tooth decay, and unaddressed dental decay can lead to further health concerns, such as gum disease, infections, and loss of teeth. In particular, young children, including those with general illnesses or disabilities and those with extensive dental needs, such as severe early childhood caries (ECC), may require DRGA to restore their oral health [2,3]. DRGA allows children to receive the necessary dental treatment without experiencing discomfort or anxiety, which can be especially important for children with dental phobia or anxiety [2,4]. By offering DRGA, pediatric dental surgeons can provide comprehensive dental rehabilitation, improve oral health outcomes, and enhance the quality of life for children with dental conditions [2,5].

Accurate scheduling of elective operations is a critical aspect of managing surgical procedures, minimizing costs, and optimizing operating room (OR) use [6,7]. Efficient use of the OR helps reduce costs for hospitals and healthcare facilities and plays a vital role in ensuring better patient outcomes and experiences [6,8]. When operations are scheduled accurately, it helps to minimize delays, cancellations, and associated costs such as wasted OR time, lost revenue, and overbooking [6,9]. Optimizing the use of OR time through accurate scheduling also helps to improve the overall satisfaction of patients, surgeons, and staff. Patients can benefit from having a better experience during surgical operations, with less stress and shorter recovery times [10]. Surgeons can better plan and prepare for procedures, resulting in more efficient use of their time and greater job satisfaction. Staff members, including nurses, anesthetists, and administrative personnel, can have more predictable workloads and schedules, contributing to job satisfaction [10]. Efficient scheduling of the OR is reliant on accurate estimation of operative time. Surgeon-estimated times (SETs) and actual operative times (AOTs) are significant metrics to evaluate OR utilization efficiency. Overestimation of surgical time may result in underutilized ORs and fewer scheduled procedures, while underestimation may lead to unplanned overtime and potential cancellation or delay of procedures [11,12]. Accurate estimation of operative time is crucial in managing surgical operations efficiently. With accurate estimates, there can be opportunities in scheduling surgical procedures, leading to longer patient waiting times, financial losses, and a need for more resources [13,14].

Despite the importance of accurate operative time estimation, it remains challenging. The estimated duration of a surgical procedure can vary significantly from the actual operative time, and studies have shown that the variation can be as much as 42% [14]. This can lead to complications such as the incorrect allocation of resources, cancellations or delays of other surgeries that are scheduled, or overbooking, which can all have significant impacts on the financial and administrative aspects of the OR’s efficient management [14,15]. Several factors contribute to the difficulty of accurately estimating operative time. Some of these factors include variations in patient physiology and anatomy, variations in the complexity of the procedure, and differences in surgeon experience and expertise [14]. Even experienced surgeons may have difficulty estimating the operative time accurately, as unexpected events or complications may arise during the surgical procedure [14]. Given the importance of accurate operative time estimation, efforts must be made to improve the accuracy of predictions [16]. It is also essential to have robust communication and collaboration between the surgical team, anesthesiologists, and supporting staff to ensure that all parties know about any changes or complications during the procedure [14,16]. These steps can ultimately lead to better patient outcomes, the more efficient use of resources, and greater satisfaction among patients, surgeons, and staff [17]. Therefore, surgeons must continuously assess their estimates for any confounding factors that may affect the duration of the procedure and strive to improve the accuracy of their predictions [18].

Collaboration between surgeons and OR staff is essential for ensuring accurate tracking of operative times [19]. OR staff are responsible for managing and monitoring the perioperative care of patients, including preparing the equipment for the surgery, managing the flow of patients, and maintaining patient safety. Surgeons rely heavily on OR staff to ensure that the surgical procedure goes smoothly [19,20]. They work together to ensure the estimated operative time is accurate and regularly updated. By working together closely, surgeons and OR staff can communicate changes or unexpected events that may result in an extension or reduction in the estimated operative time [19]. This communication can help everyone involved in the surgical procedure to adjust their plans accordingly, such as scheduling support staff, booking additional time in the OR, or rescheduling other operating procedures [19]. Accurate tracking of operative times is also crucial for analyzing and improving the surgical process [19,21]. By tracking the time taken to complete a surgical procedure and comparing it to the estimated time, the surgical team can identify areas for improvement [21]. This information enables the surgical staff to analyze past performance and adjust future surgical schedules to improve efficiency and outcomes [19,21]. Therefore, this study aimed to compare the SET provided by surgeons with the AOT in patients undergoing DRGA and to determine the influence of patient-related, surgeon-related, and anesthesia-related factors on the accuracy of the SET for the sample of DRGA. The null hypothesis for this study is that there is no significant difference between the SET and the AOT for pediatric DRGA procedures. The corresponding *p*-value for significance was less than 0.01.

## 2. Materials and Methods

A retrospective record-based analysis of SET and AOT in pediatric DRGA patients was conducted to achieve the study’s objective. The study population included all pediatric patients who underwent DRGA in a tertiary care hospital in Riyadh, Saudi Arabia between January 2022 and December 2022. This study investigated whether there is a significant difference between the SET of surgeons and the AOT in pediatric DRGA. The results of this study contribute to the field of surgical planning and patient safety by identifying the factors contributing to differences between SET and AOT in pediatric DRGA patients.

As a retrospective record-based study, the study instrument used in this study was a data collection form divided into three main sections: patient-related, surgeon-related, and anesthesia-related data. The patient-related data included age and gender, and the surgeon-related data included the surgeon’s rank and years of experience. The anesthesia-related data had time entering OR, anesthesia induction time, surgery time start and end, anesthesia recovery time, and exiting OR. The research team collected data from the hospital’s electronic medical records system of 674 patients. Convenience sampling was used to select patients who met our inclusion criteria. The inclusion criteria for the study were patients who underwent elective DRGA and pediatric patients aged less than 14 years. The exclusion criteria were patients who underwent dental surgery under local anesthesia or sedation, patients who underwent emergency dental surgery, patients with a history of medical conditions that may impact the surgery’s duration, such as bleeding disorders or cardiovascular disease, and patients with missing or incomplete medical records. Convenience sampling allowed for a quick and cost-effective way to gather data, but the findings’ generalizability to other populations may need to be improved.

A variable list was compiled for this study, which included the age and gender of patients (categorical), the rank of the surgeon (consultant or specialist; categorical), and the surgeon’s experience in years (continuous). The study also collected data on anesthesia and surgery times, including entering OR time, anesthesia induction time, surgery time start and end, anesthesia recovery time, and exiting OR time (all continuous).

This study analyzed data using descriptive statistics to summarize the data collected. The primary measure of interest was the difference between the SET and AOT for each patient. The difference in the average time between the SET and AOT was determined by comparing the central tendency of the two related groups (SET and AOT) using a statistical test. However, since the data was not normally distributed, a non-parametric alternative, such as the Wilcoxon signed rank test, was used instead. The Wilcoxon signed rank test allowed for a comparison of the mean difference between the SET and the AOT for each patient, considering the non-normality of the data. Multiple linear regression analysis was used to predict and examine the relationship between the difference in SET and AOT (dependent variable) and the various predictor variables (independent variables) identified, such as patient age and gender, surgeon rank and experience, and anesthesia induction and recovery times. Multiple linear regression was explicitly used to assess these relationships and identify which variables significantly impact the accuracy of the SET. These statistical methods allowed for a better understanding of the factors that influence the duration of DRGA in pediatric patients. Analysis was performed using IBM SPSS v26.

Ethical considerations were also considered during the study. As it was a retrospective study utilizing electronic medical and anesthesia records, participants did not obtain informed consent. However, Institutional Review Board (IRB) approval was obtained from King Saud Medical City in Riyadh (H1RI-19-Mar23-03) to ensure the study was conducted ethically and followed local regulations. The IRB reviewed the study protocol and ensured that patient confidentiality was maintained throughout the study, following ethical principles.

## 3. Results

Table 1 displays the demographic characteristics of the patient and surgeon populations involved in the study. A total of 674 patients were included, with an even distribution of male and female patients. The mean age of patients was 5.03 years, with the most common age being four years. Consultants performed most surgeries, and around 46.1% were performed by surgeons with 10–11 years of experience. The results of our study revealed a statistically significant difference between the surgeon’s estimated time and the actual operative time in pediatric DRGA (*p* < 0.0001). Therefore, we reject the null hypothesis, which states no significant difference between the two variables.

Table 2 displays that the mean difference between the SETs and AOTs (in minutes) as 19.28, with a standard deviation of 43.17, indicating a significant difference between the estimated and actual surgeries. These findings suggest that surgeons may need to adjust their estimates for pediatric DRGA to ensure adequate time for these procedures. This indicates that the surgeries were overestimated, which could have important implications for surgical planning and patient outcomes. The mean SET and AOT was 136.59 and 117.3, respectively, indicating that the average SET was higher than the AOT. Therefore, the SET needs to be more accurate and may need to be adjusted to ensure adequate time for DRGA procedures. The mean of the surgical procedure time was 84.55, with a standard deviation of 32.54, indicating that the average time for surgical procedures was around 84 min. The mean anesthesia induction and recovery time was 32.76, with a standard deviation of 8.94, indicating that the average time for anesthesia induction and recovery was around 32 min.

**Figure 1:**
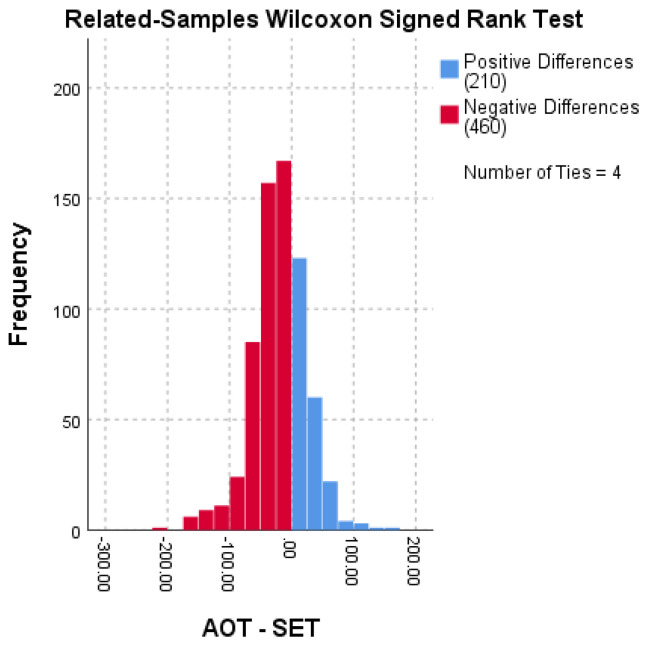
Distribution of the difference between surgeon estimated time (SET) and actual operative time (AOT) in minutes.

Figure 1 used the Wilcoxon signed-rank test to examine the difference between SET and AOT. The data were not normally distributed (N = 674), and the results showed a significant difference between the SET and the AOT (*p* < 0.0001). The median difference between the SET and the AOT was statistically significant (Mdn = 31.00, 95% CI [25.00, 36.00]). Furthermore, the data ranged from 73 to 300, with a mean of 136.59 (SD = 32.28), indicating that the SET is, on average, longer than the AOT. These results suggest that surgeons may overestimate the surgery time required, affecting surgical planning and resource allocation.

The results of the multiple linear regression analyses conducted to determine the predictors of the difference between surgeon-estimated time and actual operative time are summarized in Table 3. These results reveal that surgical procedure time is the strongest predictor, with an R square value of 0.427 and a significant *p*-value of 0.000. Years of experience with surgeons, anesthesia induction, and recovery time are also important predictors, as evidenced by their R square values and *p*-values. Age, gender, and rank of surgeons performing surgeries were not significant predictors, as evidenced by their low R square values and non-significant *p*-values.

**Figure 2:**
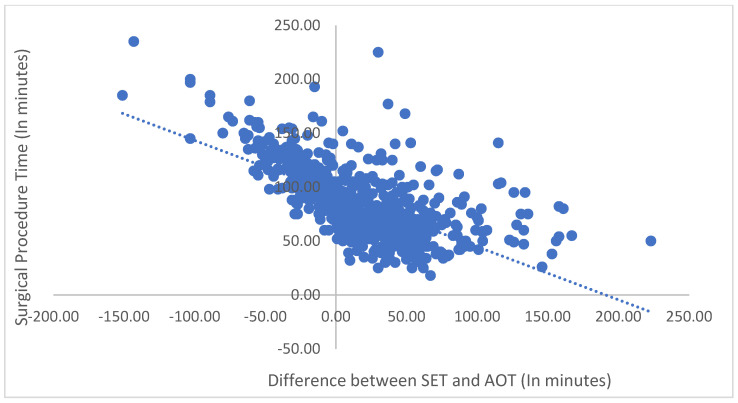
Relationship between the difference between SET and AOT and surgical procedure time (in minutes).

The scatter graph in Figure 2 illustrates the negative relationship between SET and AOT in pediatric dental procedures. Each data point represents a single procedure. The x-axis shows the difference between SET and AOT (in minutes), and the y-axis shows the total surgical procedure time (in minutes). The graph demonstrates a negative correlation between SET and AOT and the entire surgical procedure time. This indicates that procedures with a more negligible difference between SET and AOT tend to take longer. These findings are essential for improving time estimation accuracy in pediatric DRGA and optimizing patient outcomes.

**Figure 3:**
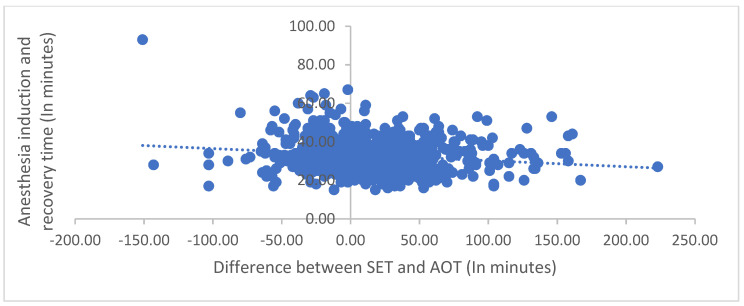
Relationship between SET and AOT and anesthesia induction and recovery time (in minutes).

The scatter graph in Figure 3 illustrates the negative relationship between SET and AOT in pediatric dental procedures with anesthesia. Each data point represents a single procedure. The x-axis shows the difference between SET and AOT (in minutes), and the y-axis shows the full anesthesia induction and recovery time (in minutes). The graph demonstrates a negative correlation between SET and AOT and the full anesthesia induction and recovery time, indicating that procedures with a more negligible difference between SET and AOT tend to have more prolonged anesthesia induction and recovery times. These findings are crucial for necessary time estimation accuracy in pediatric DRGA with anesthesia and for optimizing patient outcomes.

## 4. Discussion

Our study emphasizes the crucial importance of the accurate estimation of operative time to enhance OR efficiency and patient outcomes. Surgeons frequently overestimate case time, negatively affecting OR schedules and efficiency. To investigate the relationship between surgeon rank and the accuracy of SET, we performed a linear regression analysis, which showed no significant difference in accuracy between consultant and specialist surgeons. This overestimation can cause unplanned overtime and potential cancellations or delays of procedures. Hence, improving surgical time estimation accuracy is imperative for enhancing OR efficiency and patient outcomes. Our study revealed a significant negative relationship between SET and AOT in pediatric DRGA, with a statistically significant mean difference (*p* < 0.0001). Surgical procedure time was the strongest predictor of the difference between SET and AOT, followed by surgeon experience, anesthesia induction, and recovery time. These results are crucial for improving time estimation accuracy in pediatric DRGA and optimizing patient outcomes.

Our study on pediatric DRGA found a significant negative relationship between SET and AOT, indicating that surgeons overestimate the time required. This finding is inconsistent with a study conducted in Australia that analyzed a large dataset and found that surgeons tend to underestimate OR times for simple procedures and overestimate for complex ones. However, overall, surgeons’ predictions were reasonably accurate, with an average underestimation of only 2.3 min [22]. The authors of the Australian study recommend including surgeons’ estimates in OR scheduling to avoid the unnecessary under- or overutilization of resources [22]. Similarly, our study revealed that the strongest predictor of the difference between SET and AOT was surgical procedure time, followed by years of experience of the surgeon and anesthesia induction and recovery time. In addition to the implications for improving efficiency and reducing healthcare costs, accurate time estimation in surgical procedures is also critical for optimizing patient outcomes. Minimizing the difference between SET and AOT can reduce the risk of complications and improve patient satisfaction [6]. The negative correlation between the difference between SET and AOT and both total surgical procedure time and total anesthesia induction and recovery time, as shown in the scatter graphs, further supports this idea. Therefore, our study has important implications for improving the quality of care in pediatric DRGA and beyond [6].

This study’s results suggest that surgeons’ time overestimation can significantly impact OR scheduling difficulties. Unlike the study conducted in Australia, which found that orthopedic surgeons were the most inaccurate in predicting the necessary time, this study found that anesthetists were the most inaccurate in their time predictions, underestimating by a significant margin. However, general and plastic surgeons underestimated the time required for procedures, while orthopedic surgeons overestimated them by a small margin. These findings are consistent with previous studies that have identified the challenges associated with accurate time predictions in the OR [23,24,25]. The implications of these findings are significant for healthcare providers. By incorporating the results from this study and others, providers can implement strategies to improve scheduling efficiency, reduce healthcare costs, and optimize patient outcomes [26].

Our study found that the second most important predictor of the difference between SET and AOT following the surgical procedure time was the surgeon’s years of experience. This effect can be attributed to many factors, such as the surgeon’s familiarity with the surgical procedure, knowledge of the patient’s medical history, and the surgical environment. While data was pooled between consultant and specialist surgeons, it is possible that years of experience significantly affected estimating operation time in specialized domains. This highlights the importance of ongoing education and training for surgeons to improve their ability to estimate the time required for procedures accurately [27]. Additionally, a retrospective observational study aimed to employ OR data to provide decision support for improved OR management to overcome OR inefficiencies that may lead to access blocks and delays in treating patients requiring critical care [25]. The study used historical observations to predict long-term daily surgery caseload and developed a statistical modeling and a machine learning-based approach to estimate daily surgery demand. The study showed that daily counts of general surgery at a hospital level can be predicted with approximately 90% accuracy [25]. However, smaller subgroups of daily demands by medical specialty are less predictable. The approach can inform short-term staffing choices and long-term strategic planning [25]. Combining this with the findings from a study conducted in New Zealand that aimed to improve the accuracy of surgery duration predictions, healthcare providers can implement data-driven prediction techniques and ongoing education and training for surgeons to optimize patient outcomes, reduce healthcare costs, and improve scheduling efficiency in operating theatre procedures [24].

A study found that more accurate predictions of total procedure time can lead to increased utilization of the OR, resulting in significant financial and productivity benefits. The present study compared the SET with the AOT and found that years of surgeons’ experience, anesthesia induction, and recovery time significantly influenced the difference between SET and AOT. This highlights the importance of ongoing education and training for surgeons and anesthesiologists to improve their ability to accurately estimate the time required for procedures and optimize patient outcomes [28]. Interestingly, another study that looked at records found that accurately predicting how long anesthesia-controlled time (ACT) and surgeon-controlled time (SCT) would take was essential for efficient OR management and could lead to better use of limited OR resources [29]. The study used data from six university medical centers over seven years to support increasing SCT by 33% to account for ACT. It is clear that accurately predicting anesthesia- and surgeon-controlled time is critical for efficient OR management, and recent studies have shown the benefits of improving this prediction [29,30,31].

Both the hand surgery study and our research demonstrated that the accuracy of time estimations for surgical procedures is variable and impacted by various factors. While the hand surgery study identified surgeon, procedure type, and surgical time as significant predictors, our study found that surgical procedure time was the most influential factor, followed by surgeon experience, anesthesia induction, and recovery time. These findings highlight the importance of considering multiple variables when estimating procedure times accurately [32,33].

Our study identified surgical procedure time as the strongest predictor of SET accuracy. In contrast, the other study focused on developing statistical models using linear regression and supervised machine learning to predict surgical time duration. The results of this study showed that the machine learning algorithm had the highest predictive capability, with the surgeon-specific model being superior to the service-specific model [34]. By identifying factors impacting surgical time estimations and developing statistical models to predict surgical time duration, surgeons and OR managers can collaborate to enhance scheduling and OR efficiency, leading to better patient outcomes and increased productivity [34].

While this single-center record-based retrospective observational study provided valuable insights into the relationship between the variables under investigation, it was essential to acknowledge the potential limitations of the study design. One possible limitation was the risk of missing or incomplete data, which could have affected the accuracy and reliability of the study results. Additionally, the quality and consistency of the data may have varied across different sources, which could have introduced bias or variability in the results. Finally, the study design may have limited our ability to establish causal relationships between variables or draw definitive conclusions about the underlying mechanisms driving the observed results.

In summary, our study aimed to determine the difference between SET and AOT. We found a significant difference between the two, with surgeons tending to overestimate case time. This discrepancy can lead to underutilization in the OR schedule and decreased efficiency. While our study has several limitations, including a relatively small sample size and a single-center design, it provides a foundation for future research to build upon and improve case time estimation accuracy. Future studies should replicate our findings in more extensive, multi-center studies and explore other factors impacting case time estimation accuracy. Additionally, developing and validating statistical models to predict case time duration that can be easily integrated into existing electronic medical record systems would provide a practical solution for improving OR efficiency and scheduling.

## 5. Conclusions

This study’s findings emphasize the importance of accurately estimating operative times in surgical procedures. The considerable difference between the SET and the AOT required for surgery could negatively impact patient outcomes, leading to dissatisfaction. This difference could also lead to delays and inefficiencies in the OR schedule, decreasing productivity and resource allocation. Our findings also highlight the importance of considering the complexity of the procedure when estimating the surgical time. Complex operations require more attention and might need more time than anticipated. Surgeons should be aware of this and make sure that they estimate more appropriately, considering the procedure’s complexity.

Improving the accuracy of surgical time estimation can positively impact patient outcomes, increase OR efficiency, and increase patient satisfaction, thus potentially reducing costs for healthcare organizations. As such, it is crucial to identify the factors that impact case time estimation accuracy and develop more precise estimation methods to reduce the discrepancy between the SET and the AOT. Our study highlights that accurate estimation of operative time is necessary for the efficient scheduling of the OR and for optimal patient outcomes. Improving the accuracy of surgical time estimation methods can reduce delays, enhance surgical team productivity, and increase patient and healthcare providers’ satisfaction. Further research must be conducted to develop reliable methods to improve case time estimation accuracy and optimize the use of OR resources.

## Figures and Tables

**Table 1:** Demographic characteristics of patients and surgeons involved in the study.

Age Distribution of Patients	
Age	Frequency (%)
2	45 (6.70)
3	106 (15.70)
4	163 (24.20)
5	145 (21.50)
6	78 (11.60)
7	50 (7.40)
8	41 (6.10)
9	26 (3.90)
10	5 (0.70)
11	8 (1.20)
12	6 (0.90)
13	1 (0.10)
Total	674 (100)
**Gender Distribution of Patients**	
**Gender**	**Frequency (%)**
Female	326 (48.40)
Male	348 (51.60)
Total	674 (100)
**The Rank of Surgeons Performing Surgeries**	
**Rank**	**Frequency (%)**
Consultant	379 (56.20)
Specialist	295 (43.80)
Total	674 (100)
**Years of Experience with Surgeons Performing Surgeries**	
**Years**	**Frequency (%)**
10	158 (23.40)
11	153 (22.70)
20	110 (16.30)
24	74 (11)
28	68 (10.10)
31	111 (16.50)
Total	674 (100)

**Table 2:** Comparison of estimated and actual operative times for surgical procedures.

	SET *	AOT *	Difference between the SET and AOT *	Surgical Procedure Time *	Anesthesia Induction and Recover Time *
N	674	674	674	674	674
Mean	136.59	117.30	19.2834	84.55	32.76
Std. Error of Mean	1.24	1.30	1.66	1.25	0.34
Median	126.00	114.00	17.00	80.50	31.00
Mode	120.00	122.00	8.00	60.00	30.00
Std. Deviation	32.29	33.64	43.17	32.54	8.94
Variance	1042.59	1131.80	1863.78	1058.61	79.95
Range	227.00	228.00	374.00	217.00	78.00
Minimum	73.00	50.00	−151.00	18.00	15.00
Maximum	300.00	278.00	223.00	235.00	93.00
Sum	92,061.00	79,064.00	12,997.00	56,987.00	22,077.00

SET: Surgeon Estimated Time, AOT: Actual Operative Time, * (In minutes).

**Table 3:** Multiple linear regression analysis of predictors of the difference between.

Model	R Square	Standard Error of the Estimate	*p* Value
Age of Patients	0.002	43.16437	0.269
Gender of Patients	0.002	43.16091	0.249
Rank of Surgeons	0.002	43.16757	0.289
Years of experience as surgeons	0.035	42.45082	0.000
Anesthesia Induction and Recovery Time	0.023	42.70586	0.000
Surgical Procedure Time	0.427	32.69848	0.000

## Data Availability

The principles of open science and transparency in research are fully supported, and the data supporting the reported results in this study are available upon request from the corresponding author. No new datasets were generated during this study, and all data used in this research were obtained from publicly available sources or with appropriate permissions from the original data owners. The article will clearly state any data that cannot be publicly available due to privacy or ethical restrictions. The MDPI Research Data Policies have been followed.
